# Characterization of uterine involution using B-mode ultrasonography, color Doppler and elastography (acoustic radiation force impulse) for assessing postpartum in Santa Inês ewes

**DOI:** 10.1590/1984-3143-AR2022-0110

**Published:** 2023-06-30

**Authors:** Renata Sitta Gomes Mariano, Victor José Correia Santos, Augusto Ryonosuke Taira, Priscila Del Aguila da Silva, Mariana Garcia Kako Rodriguez, Luciana Cristina Padilha-Nakaghi, Ana Paula Rodrigues Simões, Marjury Cristina Maronezi, Michelle Lopes Avante, Ricardo Andres Ramirez Uscategui, Bruna Bressianini Lima, Marcus Antônio Rossi Feliciano, Pedro Paulo Maia Teixeira, Welter Ricardo Russiano Vicente

**Affiliations:** 1 Departamento de Patologia, Reprodução e Saúde Única, Faculdade de Ciências Agrárias e Veterinárias, Universidade Estadual Paulista “Júlio de Mesquita Filho”, Jaboticabal, SP, Brasil; 2 Departamento de Clínica e Cirurgia Veterinária, Faculdade de Ciências Agrárias e Veterinárias, Universidade Estadual Paulista “Júlio de Mesquita Filho”, Jaboticabal, SP, Brasil; 3 Departamento de Medicina Veterinária, Faculdade de Zootecnia e Engenharia de Alimentos, Universidade de São Paulo, Pirassununga, SP, Brasil; 4 Departamento de Medicina Veterinária, Universidade Federal do Pará, Castanhal, PA, Brasil

**Keywords:** puerperium, uterine involution, uterine stiffness, sheep

## Abstract

The aim of this study was to investigate uterine involution using ultrasonography techniques during postpartum. Postpartum ultrasonography evaluation (B-mode, color Doppler and Acoustic Radiation Force Impulse elastography) of the uterus was performed by transabdominal approach at immediate after birth and sequentially every 48 hours, during 30 days. The uterine echotexture did not present significant variations (*P* >0.05) being homogeneous in most evaluations; echogenicity of the uterus increased along the evaluation period (*P* =0.0452). Progressive and remarkable decrease of the total uterine diameter (UD) were observed (*P* <0.0001), especially during the first days postpartum. The thickness of uterine wall gradually decreased, as well the endometrial, myometrium and lumen diameters (*P* <0.0001). Uterine blood flow was assessed by Doppler and decreased during postpartum period, being significantly lower (*P*=0.0225) on the 30^th^ day of postpartum. Uterine parenchyma presented as homogeneous dark areas (not deformable) on qualitative ultrasound elastography and the means shear velocity values of the uterine wall on quantitative elastography did not differ. This is the first study that evaluate the stiffness of uterine wall in healthy ewes, providing baseline data about quantitative and qualitative stiffness of the normal uterus, and it may be a useful tool for early diagnosis of uterine alterations during the postpartum period, using the reference parameter established for the assessment of uterine integrity during postpartum period.

## Introduction

The placenta in sheep originates through stimulation of the vascular allantois and the chorion avascular, which allows a greater contact surface of the mother and the fetus through the development of projections from the villi, which are together in different areas, called cotyledons. Thus, the placenta of sheep can be classified as zonary, cotyledonary and synepitheliochorial (Almeida, 2019).

The post-partum or puerperium is characterized by many physiological changes in the uterus occurring after delivery (Simões, 2019). The process of uterine involution involves intense modifications as contraction of muscle fibers, catabolism, physical shrinkage, necrosis, sloughing of the uterine caruncles and regeneration of the uterine epithelium (Bajcsy et al., 2005; Sheldon et al., 2008). During involution, the size of the uterus reduces, both the myometrium and endometrium are structurally restored, and the uterus will be prepared for a next conception (Sheldon, 2004).

Postpartum fertility in ewes depends on uterine physiological involution and restoration of cyclicity. However, few information is available on the reestablishment between the time of complete uterine involution and the return to cyclical activity (Nasciutti et al., 2001; Hayder and Ali, 2008). The physiological events that take place during the postpartum period influence next conception and pregnancy and reveal its reproductive and economic relevance. Therefore, it is important to know the physiological postpartum uterine regression in detail (Hauser and Bostedt, 2002) to achieve a satisfactory interval between parturition (Sánchez et al., 2002). Thus, we believe monitoring postpartum period allows an early diagnosis of alterations, and an efficient treatment of uterine diseases, to limit their negative effects on fertility.

Ultrasonography may reveal details of the progressive changes in the uterus of small ruminants (Hauser and Bostedt, 2002; Badawi et al., 2014), plays a key role to distinguish normal from abnormal postpartum uterus, and allows for an objective measurement and visualization of the uterine horns and lumen diameter (Medan and El-Daek, 2015). Advantage of using ultrasonography to monitor uterine involution is that they can potentially be applied under practical conditions, allowing analysis of either physiological or pathological events (Bajcsy et al., 2005; Teixeira, 2017).

According to Elmetwally and Bollwein (2017) Doppler ultrasound imaging can be used successfully to evaluate hemodynamic changes in the uterine vasculature during the early postpartum period in sheep and goats. They also highlight that the hemodynamic changes in uterine blood flow during the early postpartum period can be used to assess resumption of ovarian follicular activities and uterine involution during the postpartum period in sheep and goats.

Elastography by acoustic radiation force impulse imaging (ARFI), a technique based on ultrasonography has been used to evaluate stiffness in a tissue using a short acoustic push pulse in the target tissue (Karaman et al., 2016). It is considered a way to “imaging palpation” (Woźniak et al., 2016).

ARFI elastography is now emerging as a mainstream tool for ultrasound-based diagnosis. The advantages of elastography include the repeatability of objective measurements, and the ability to evaluate qualitative and quantitative information of tissue stiffness without requirement for external compression (Sporea et al., 2012; Alan et al., 2016). The elasticity of soft tissues is measured to investigate differential diagnosis of many diseases, such as inflammation, fibrosis, and tumoral tissues (Tan et al., 2013; Karaman et al., 2016). However, there are no elastographic studies of uterus during postpartum involution in ewes using the ARFI technique, which could provide relevant physiological and pathological information.

Considering the necessity to carry out studies to establish the physiological patterns of postpartum that enable early diagnosis methods for evaluation of possible puerperal changes in ewes, monitoring this period becomes essential. This prevents a decline in reproductive efficiency, infertility and delay in the return to cyclicity. Consequently, the aim of this study was to describe the physiological changes throughout uterine involution, evaluating the uterine regression and tissue stiffness during postpartum by means of B-mode ultrasonography, color Doppler and ARFI elastography to elucidate the mechanism of physiological uterine involution and uterine characterization during the postpartum period in ewe.

## Methods

### Experimental protocol

The study was performed during the months of September and November of 2016. Twenty adult multiparous healthy Santa Ines ewes, aged 3.1±1.1 years, weighing 45.4±4.3 kg (before conceiving) and exhibiting a mean body score of 3 (scale 1-5,) (Jefferies, 1961), were selected for this study after checking non-intercurrent full-term normal delivery (singleton gestation only), and normality at physical examination, complete blood count, plasma concentrations of total proteins and fibrinogen, and ultrasonography of the reproductive system (Silva et al., 2019).

Ewes were brought to the ultrasound laboratory, maintained in standing position, without pharmacology restraint. A wide trichotomy of the abdominal region was performed and coupling gel applied. The ultrasonographic evaluation (B-mode, Doppler and elastography) of the uterus was performed as follows: immediate after delivery (M0) and sequentially every 48 hours during the first 30 days postpartum (DPP) totalizing 16 experimental samples per animal.

### B-mode and color Doppler ultrasonography

Ultrasonography evaluation started on B-mode using the ACUSON S2000® (Siemens, Munich, Germany) ultrasound system and were performed by the same experienced operator using a convex multi-frequential transducer (4C1; 4.0 MHz; Siemens, Munich, Germany) transabdominal. The transducer was positioned on the right or left inguinal area (Silva, 2021). Ultrasound examination began by locating the bladder, which were used as an acoustic window to facilitate examination, especially after the first week postpartum (Ioannidi et al., 2017). Images were obtained on the longitudinal and the transverse ultrasonographic planes. When uterus was located, the qualitative characteristics echotexture (homogeneous or heterogeneous), echogenicity (hypoechoic, hyperechoic or isoechoic compared the adjacent tissues), and the characteristics of its contents (anechoic, hypoechoic, hyperechoic; with or without signs of cellular debris) were evaluated. The thickness of the entire uterine wall, thickness of myometrium and endometrium, diameter of the total uterine body and diameter of its lumen were measured (mm) in this moment.

Subsequently, color Doppler was activated and used to determine the characteristics of uterine blood flow: vascularization (presence or absence), type of flow (arterial, venous or mixed) and vessels localization (peripheral, central, or diffuse), during the same postpartum period

### ARFI elastography

During the B-mode exam, once the structures are located and measured, a specific module Virtual Touch Tissue Quantification-VTTQ (Siemens, Munich, Germany) designed for qualitative and quantitative image analyses was activated. Initially, this software provides grayscale image known as elastography in which the qualitative characteristics of tissue elasticity were evaluated according to: the shades, dark shades (black) indicate rigid tissues (less elastic), while light shades (white) indicate soft tissues (more elastic); homogeneous or heterogeneous; and deformable or non-deformable.

After qualitative analysis, in the same image, the quantitative analysis module (Acoustic Radiation Force Image-ARFI; Siemens, Munich, Germany) was activated, with this module it is necessary to sample regions of interest, using predefined calipers, which were randomly positioned in three different areas of the uterine wall (endometrium/myometrium), at a depth ranging from 0.5 to 5.0 cm in each portion evaluated, according to the anatomical topography, after this procedure the software automatically provides the shear wave velocity (SWV) expressed in m/s, the average of three regions measurement was used to calculate the uterine wall SWV.

### Statistical analysis

This is a prospective observational study with repeated measures. Statistical analysis was performed using the software R, version 3.3.0 (R® foundation for statistical computing, Austria). Data were tested initially for normality (Shapiro test) and homoscedasticity of variances (Barlett test). The quantitative variables resulting from ultrasound examination were correlated with the evaluated moments by Pearson test, compared between these same moments by the analysis of variance and if it was significant, orthogonal contrasts were tested to evaluate the behavior of the variable adjusted to regression models (linear, quadratic, and cubic). For qualitative variables, the proportion of characteristics resulting from qualitative B-mode, doppler and elastography evaluations were compared, using multiple Fisher's exact tests. The level of significance was set at 5% for all tests (P<0.05), quantitative results are presented as the mean ± SD (standard deviation) and qualitative results as proportion of characteristics (%).

### Ethical and animal aspects

All animal procedures were approved by the Animal Ethics and Welfare Committee of the Faculty of Agricultural and Veterinary Sciences, Univ. Estadual Paulista (Unesp), Jaboticabal, Brazil (protocol N ° 12338/15). The ewes were kept in an elevated sheep house at the Animal Reproduction Department, fed with corn silage, balanced commercial concentrate, mineral salt and water *ad libitum* during pregnancy and throughout the postpartum period

## Results

Uterine ultrasonography could be performed in all animals (*n*=20), and the uterine characteristics were determined without any difficulties by transabdominal approach. No intercurrences were observed during the experimental period and no signs of discomfort during exams were also observed.

Uterine echogenicity varied during postpartum involution process (P=0.0452) from the 10^th^ day where 45% of the animals presented hyperechoic uterine wall, 45% hypoechoic and 10% isoechoic, until 20^th^ day, after which the majority (67-82%) was isoechoic and 18 to 72% was hypoechoic. The echotexture did not present variations (*P*>0.05) remaining homogeneous throughout the postpartum days (76-100%; Table 1).

**Table 1:** Rate of qualitative variables (%) evaluated by different ultrasonography methods (B-mode and Doppler) of uterine structure evaluated at different postpartum days (DPP) in healthy Santa Inês ewes.

**DPP**		**0**	**2**	**4**	**6**	**8**	**10**	**12**	**14**	**16**	**18**	**20**	**22**	**24**	**26**	**28**	**30**
**Qualitative parameters**		**%**	**%**	**%**	**%**	**%**	**%**	**%**	**%**	**%**	**%**	**%**	**%**	**%**	**%**	**%**	**%**
Uterine echogenicity	Hyperechoic	4.5	4.5	14.3	9.1	45.5	45.0	5.0	5.0	4.8	0.0	15.0	0.0	0.0	0.0	0.0	0.0
	Isoechoic	81.8	72.8	66.7	86.4	9.0	10.0	55.0	60.0	61.9	66.7	67.0	61.5	50.0	82.4	66.7	72.2
	Hypoechoic	13.7	22.7	19.0	4.5	45.5	45.0	40.0	35.0	33.3	33.3	18.0	38.5	50.0	17.6	33.3	27.8
Uterine echotexture	Homogeneous	76.2	86.4	90.5	100.0	86.4	100.0	100.0	95.0	95.2	88.9	88.9	84.6	95.0	94.1	80.0	100.0
	Heterogeneous	23.8	13.6	9.5	0.0	13.6	0.0	0.0	5.0	4.8	11.1	11.1	15.4	5.0	5.9	20.0	0.0
Cellular debris		31.8	50.0	33.3	36.4	27.3	20.0	0.0	10.5	4.8	0.0	5.6	0.0	5.0	0.0	0.0	0.0
Content echogenicity	Hyperechoic	31.8	13.6	9.5	4.5	0.0	0.0	0.0	0.0	0.0	0.0	0.0	0.0	0.0	0.0	0.0	0.0
	Hypoechoic	18.2	36.4	33.3	18.2	18.2	0.0	0.0	0.0	0.0	0.0	0.0	0.0	0.0	0.0	0.0	0.0
	Anecoic	50.0	50.0	57.2	77.3	81.8	100.0	100.0	100.0	100.0	100.0	100.0	100.0	100.0	100.0	100.0	100.0
Vascularization		100.0	100.0	100.0	95.5	100.0	100.0	100.0	100.0	100.0	88.9	94.4	92.3	100.0	88.2	86.7	50.0
Flow type	Mixed	45.5	50.0	47.6	76.2	68.2	70.0	73.7	68.4	57.1	62.5	70.6	75.0	85.0	66.7	76.9	55.6
	Arterial	54.5	50.0	52.4	23.8	31.8	30.0	26.3	31.6	42.9	37.5	23.5	25.0	15.0	33.3	23.1	44.4
	Venous	0.0	0.0	0.0	0.0	0.0	0.0	0.0	0.0	0.0	0.0	5.9	0.0	0.0	0.0	0.0	0.0
Vessels localization	Peripheral	72.7	95.5	95.2	100.0	90.9	95.0	100.0	100.0	100.0	100.0	100.0	100.0	100.0	100.0	100.0	100.0
	Central	0.0	0.0	4.8	0.0	0.0	0.0	0.0	0.0	0.0	0.0	0.0	0.0	0.0	0.0	0.0	0.0
	Diffuse	27.3	4.5	0.0	0.0	9.1	5.0	0.0	0.0	0.0	0.0	0.0	0.0	0.0	0.0	0.0	0.0

**Figure 1:**
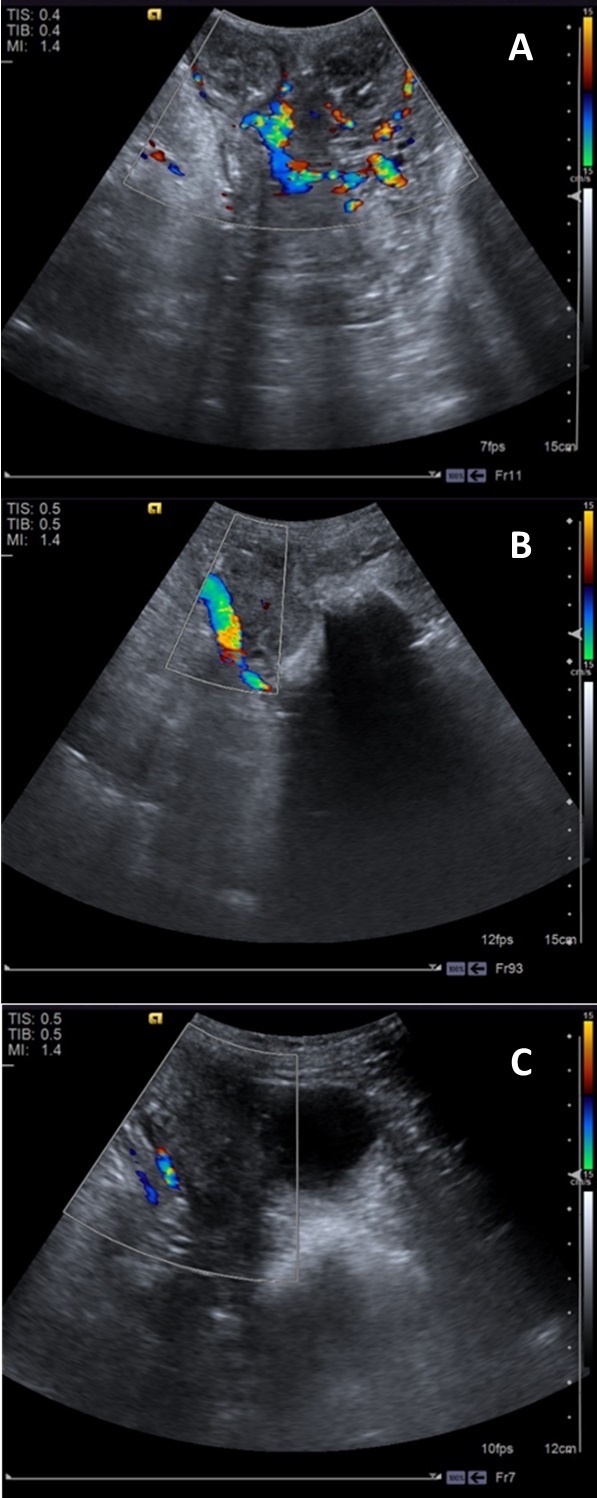
Ultrasound color Doppler image of uterine blood flow during postpartum in healthy ewes. **Figure 1A,**11C: 4, 18 and 30 DPP, respectively. Vessels in red are those that approach the transducer, that is, flow in the proximal direction; the vessels in blue are those that move away from the transducer, that is, flow in the distal direction. Mixed color images are areas of blood turbulence.

**Figure 2:**
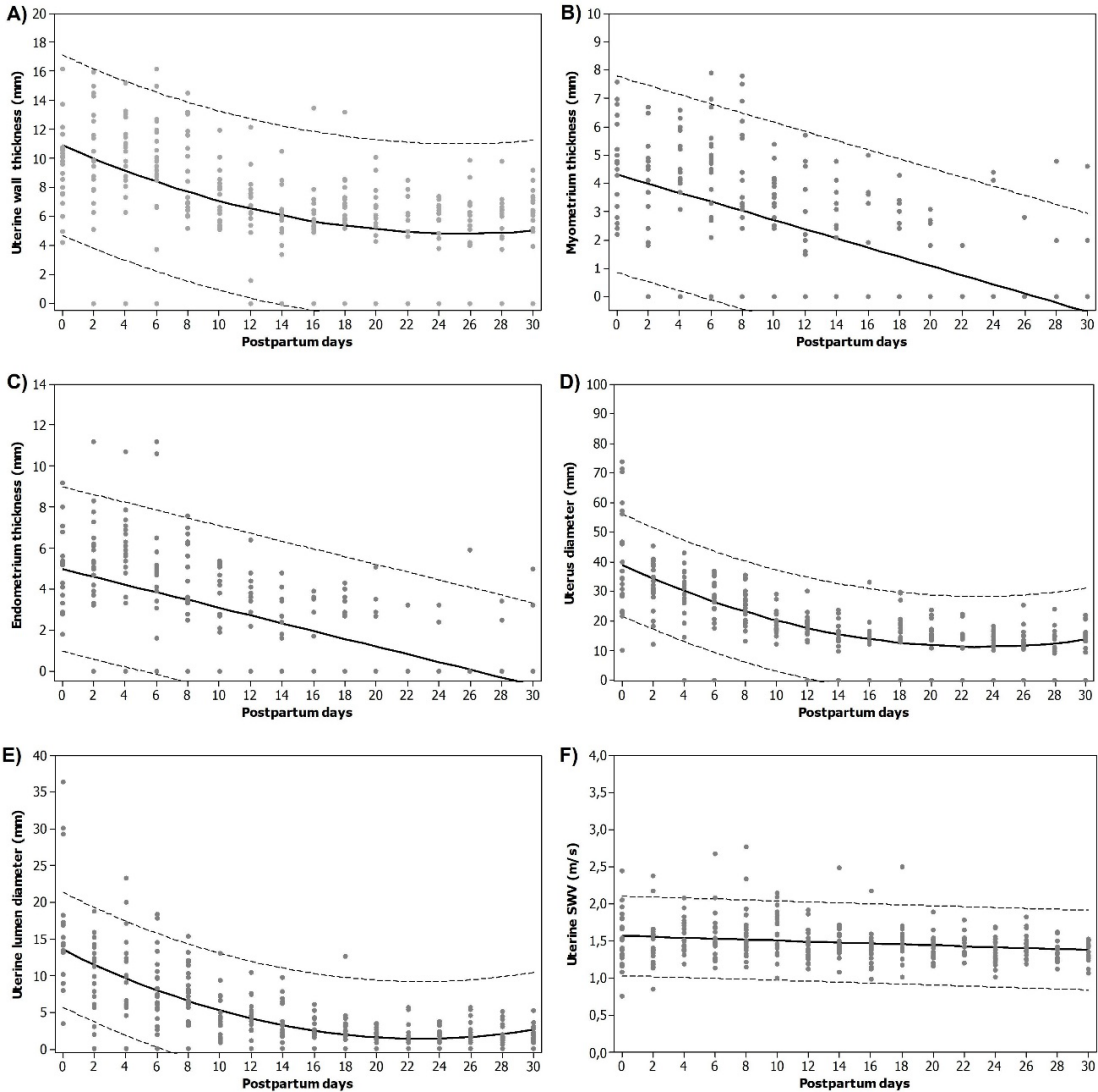
**Graphic ill**ustration showing the relation between B-mode uterine measurements, uterine shear wave velocity (SWV) electrography evaluation and Postpartum days. The dots indicate the collected values, the black line the regression that explains the behavior and the dotted line the 95% confinement margin.

Regarding color Doppler evaluation, the uterus vascularization was less evident after 18th day, and lower on the 30th day (*P*= 0.0225; Figure 1B), and highly vascularized in 100% of animals, and the blood flow was easily notable in the first days after parturition (Figure 2A). The type of flow (arterial and venous), that was mostly mixed (> 50%) and the vessels localization peripheral (> 90%) without variations during evaluation period (P>0.05; Table 1).

The uterine content (lochia) was present in 100% of animals until 8^th^ day postpartum, decreased its proportion gradually (P=0.0215) until the 22^nd^ day in which no content was observed in any of the animals. This uterine content was hyperechoic (32%) and hypoechogenic (18%) at the first day postpartum, becoming anechoic in 100% of animals from the 10^th^ day (P=0.0335), and with cellular debris always less than 20% from the 10th day (Table 1).

The thickness of the uterine wall gradually decreased (P <0.001; r = –0.501; Table 2) with the advancement of postpartum days, falling significantly after 6 days and corresponding to a quadratic regression model (Uterine wall = 10.92 – 0.4747*DPP + 0,009266*DPP^2^; R^2^ = 0.87; Figure 2). The thickness of the myometrium gradually decreased (P <0.0001; r = –0.645; Table 2) with the advancement of days, the decrease being significant after 12 days and becoming almost imperceptible after 20 days, its behavior was explained by a linear model (myometrium = 4.322 – 0.1621*DPP; R^2^ = 0.85; Figure 1). The thickness of the endometrium gradually decreased (P <0.0001; r = –0.648; Table 2) with the advancement of days, with a significant drop after 10 days and becoming almost imperceptible after 24 days and its behavior explained by a linear model (Endometrium = 4.984 – 0.1891*DPP; R^2^ = 0.85; Figure 2).

**Table 2:** Mean values ± Standard deviation (SD) of measurements of the uterine regions (mm), shear wave velocities (SWV – m/s) and depth (cm) in different days postpartum (DPP) in healthy ewes.

**Variable**	**Uterus diameter**		**Uterine wall**		**Myometrium**		**Endometrium**		**Lumen diameter**		**SWV**		**Depth**	
**DPP**	**Mean**	**SD**	**Mean**	**SD**	**Mean**	**SD**	**Mean**	**SD**	**Mean**	**SD**	**Mean**	**SD**	**Mean**	**SD**
**0**	39.9	18.0	10.2	5.5	3.8	2.2	4.1	2.5	12.1	10.5	1.5	0.4	3.5	1.2
**2**	32.3	8.2	9.9	3.7	3.5	2.2	4.7	2.9	9.1	5.5	1.4	0.3	3.3	1.5
**4**	30.0	10.0	10.2	3.3	4.2	2.0	5.2	2.7	9.1	6.2	1.6	0.2	2.9	1.8
**6**	27.5	8.2	9.7	3.5	4.3	2.0	4.7	2.6	7.7	5.1	1.6	0.3	2.8	1.5
**8**	25.4	6.2	8.9	2.9	3.8	2.2	4.0	2.2	7.7	3.7	1.6	0.4	2.8	1.7
**10**	19.3	4.2	7.4	1.8	2.5	1.8	2.8	2.1	4.5	3.2	1.6	0.3	2.5	0.8
**12**	16.3	7.9	6.0	3.3	1.6	1.9	1.8	2.2	3.8	2.8	1.4	0.2	2.6	0.9
**14**	13.8	7.1	4.9	3.0	1.6	1.8	1.5	1.7	3.4	2.7	1.5	0.3	2.9	0.9
**16**	10.8	8.9	4.3	3.6	0.8	1.6	0.9	1.6	2.0	2.0	1.4	0.2	3.5	1.1
**18**	17.7	6.8	6.3	2.9	1.2	1.6	1.3	1.7	3.2	2.6	1.5	0.3	3.1	0.9
**20**	13.5	7.0	5.6	2.9	0.6	1.1	0.8	1.6	1.7	1.3	1.4	0.2	2.7	0.7
**22**	10.6	8.1	4.4	3.2	0.1	0.5	0.2	0.9	1.8	1.9	1.4	0.2	2.7	1.3
**24**	9.9	6.9	4.2	3.0	0.4	1.3	0.3	0.9	1.4	1.2	1.4	0.2	3.3	1.7
**26**	11.4	7.4	4.7	3.1	0.2	0.7	0.3	1.4	1.9	1.9	1.4	0.2	3.1	1.3
**28**	12.5	6.3	4.9	2.9	0.4	1.3	0.4	1.0	2.0	1.6	1.4	0.2	2.7	1.2
**30**	13.8	5.9	5.8	2.5	0.4	1.2	0.5	1.4	2.0	1.3	1.4	0.1	3.0	1.2

The diameter of the uterus gradually decreased (P <0.0001; r = –0.645; Table 2) with the advancement of the postpartum days, falling significantly after 8 days and its behavior being explained by a quadratic model (UD = 38.93 – 2.400*DPP + 0.05191*DPP^2^; R^2^ = 0.96; Figure 2). The diameter of the uterine lumen also decreased gradually (P = 0.0002; r = –0.612; Table 2) as the days progressed, falling significantly after 8 days and its behavior was explained by a quadratic model (uterine lumen = 13.53 – 1.058*DPP + 0.02313*DPP^2^; R^2^ = 0.95; Figure 2).

**Figure 3:**
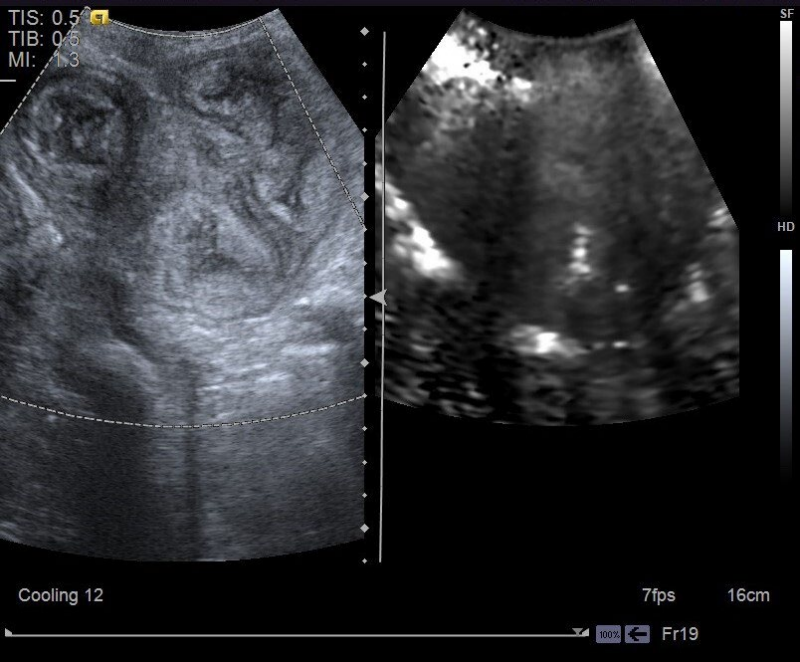
Qualitative uterine ARFI elastography analysis during postpartum. Note the B-mode image (left) and the image of the elastography (right) of the uterus showing a homogeneous and dark (hard) image, respectively.

**Figure 4:**
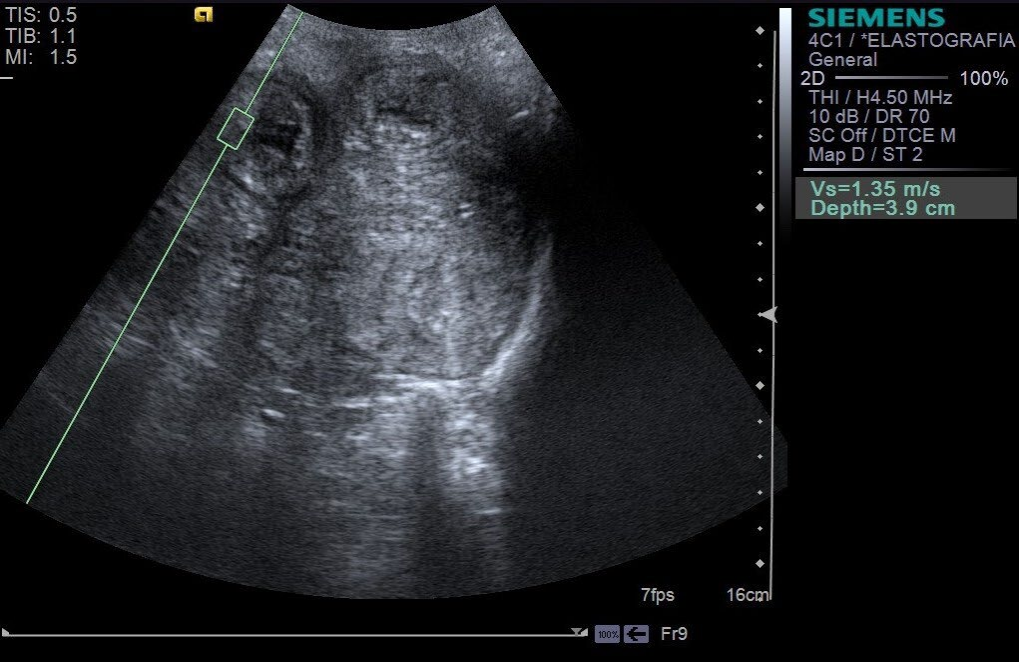
Quantitative uterine ARFI elastography analysis during postpartum. Note the measurement of the shear velocity of uterine wall (Vs= 1.35 m/s), with the presence of a caliper for the portion assessed.

Based on the qualitative elastography examination of the uterine tissues, 100% of the animals showed appearance of hard uterine tissue (darker), meaning were not deformable during uterine involution (Figure 3). Of the elastograms 84% were classified as homogeneous (Figure 3) and 16% heterogeneous, however no correlation to postpartum moments was observed (*P*=0.9010). The uterine SWV were constant during postpartum assessment (*P*=0.2176; r = –0.210; Table 2), according to measurement of the shear velocity of uterine wall, with the presence of a caliper for the portion assessed (Figure 4).

## Discussion

In this study, we showed the efficacy and the executability of B-mode, color Doppler and ARFI elastography as a safe and non-invasive method for evaluating the assessment of physiological uterine involution during postpartum in ewes. It was established reference data for values of uterine biometry, uterine wall stiffness and blood flow in healthy animals during this period.

Shortly after the moment of the partum, there are several physiological uterine modifications for the return of the uterus to enter in a new reproductive cycle (Elmetwally and Bollwein, 2017), such as degeneration of the caruncles, reduction in the size of the uterus and recovery of myometrium and endometrium (Bajcsy et al., 2005). Initially, the uterine wall is covered with caruncles (Van Wyk et al., 1972b), which could physiologically explain the increase in echogenicity of the uterine wall in B-mode found in this study. In addition, the echogenicity of the uterus may be related to uterine tone and hormonal changes (Degefa, 2003; Viñoles-Gil et al., 2010).

Previous studies in small ruminants reported difficulties to observe caruncles (Kähn, 2004; Ababneh and Degefa, 2005; Fasulkov, 2014; Zongo et al., 2015) and differentiation from uterine layers (Hauser and Bostedt, 2002) by ultrasound around 15 days postpartum, reflecting the images found in this study of hypoechogenicity and isoechogenicity, and no more hyperechoic images after 20 DPP.

### B-mode ultrasonography

A study using B-mode ultrasonography to evaluate uterine regression in ewes reported that a delay of the separation process of the fetal membranes from the caruncles is characterized by a hyperechoic border of the caruncles with the central site more hypoechoic (Hauser and Bostedt, 2002). It also supports our results that the variation in the pattern found in this study may reveal an alteration in the normal course of development of uterine involution.

Regarding to B-mode echotexture, a similarity of echotexture in perimetrium and myometrium was observed in ewes (Hauser and Bostedt, 2002). As well as similar echotextures of caruncles and the endometrium in goats (Zongo et al., 2015), making it difficult the differentiation by ultrasonography during the first weeks of postpartum, corroborating to our findings that the parenchymal echotexture did not present significant variations remaining homogeneous.

In the present study, the uterine contents were present and hypoechogenic at the first days postpartum, corroborating with findings described by Hauser and Bostedt (2002), Ababneh and Degefa (2005) and Badawi et al. (2014), and it decreased gradually by the 22^nd^ day postpartum, when it was no longer observed. This finding corresponds to the same reported by Van Wyk et al. (1972b), only little amounts of fluid in the uterus up to day 20 pp. The debris observed during the initial days pp. in the uterine lumen may also be tissues, debris and blood normally present within the uterus during the postpartum (Shen et al., 2003). Hauser and Bostedt (2002) suggested that little amounts of fluids seem to be physiological in postpartum uterus.

A remarkable reduction in the UD was observed during the first DPP, however, after this period the reduction was decreased. Similar results were reported in ewes by Ioannidi et al. (2017), Elmetwally and Bollwein (2017), Zduńczyk et al. (2004), and Hauser and Bostedt (2002). Ultrasonography revealed to be a useful and reliable method to observe the uterine involution in sheep.

Indeed, in our study it was possible to evaluate the measurements of thickness of myometrium, endometrium and the biometry of uterine body. The thickness of uterine wall, myometrium and endometrium decreased over time, which may be related to the uterine contractility during the early postpartum period, and the regeneration of the uterine layers (Ioannidi et al., 2017). The uterine wall thickness gradually decreased, and reduced significantly at the 6^th^ day pp., in accordance to data reported by Fasulkov (2012), who observed significant differences in the uterine wall thickness by the 9^th^ day postpartum in goats.

### Doppler ultrasonography

The present study presents reference data on the changes in uterine blood flow during the postpartum period in healthy Santa Ines ewes, assessed by color Doppler ultrasonography during the uterine involution process. Our results agreed with findings in ewes, whereupon the uterine blood flow decreased during the postpartum period (Elmetwally et al., 2016; Elmetwally and Bollwein, 2017). This reduction has been associated with the great decrease in the uterine size after parturition and may be related to the important uterine blood flow changes right after the partum, due to reduction of the metabolic requirements for pregnancy (Elmetwally and Bollwein, 2017). Similar results were reported in cows (Heppelmann et al., 2013), mares (Lemes et al., 2017), as well as in women during the initial postpartum (Van Schoubroeck et al., 2004).

### ARFI elastography

According to our knowledge, studies reporting the use of ARFI elastography for evaluation of changes in uterine stiffness during postpartum involution have not been reported in animals. However, a study conducted by Tanaka et al. (2011) in humans evaluated the stiffness of the uterus and cervix before, immediately after and 1 and 2 h after placental delivery, while our study assessed the uterus for 30 days. Additionally, the repeatability of the procedure, every 48h, highlights the importance and the consistence of the presented data in this study, as well as proves that the elastography ARFI is not harmful.

In this study, the uterine parenchyma exhibited as rigid (dark) and homogeneous tissue indicating that was not easily deformable. This is consistent with data of a recent study conducted by Frank et al. (2016) in the evaluation of normal uterine tissue in non-pregnant women, emphasizing that normal uterine tissue is relatively homogeneous. From the uterine characterization in the physiological puerperium, it reinforces the suggestion that a different elastographic pattern is potentially indicative of alterations, pathological or not, in the uterine parenchyma.

Shear wave elastography is based on the concept that shear waves move faster through more rigid regions in a tissue. It has been used successfully in placenta of normal and pre-eclamptic pregnancies in women (Cimsit et al., 2015; Kılıç et al., 2015; Wu et al., 2016) and in the evaluation of cervix in pregnant women (Gennisson et al., 2011; Carlson et al., 2014; Hernandez-Andrade et al., 2014). Additionally, SWV was considered a valuable method to objectively quantify the cervical stiffness and as a complementary diagnostic tool for preterm birth and for labor induction success in pregnant sheep as animal model (Peralta et al., 2015). In our study, similarly to the qualitative results that demonstrated a consistent pattern of uterine wall stiffness, the quantitative data (SWV) were also constant in relation to the elasticity of the uterine wall in the involution process.

This is, to the authors’ knowledge, the first study that have been described in veterinary medicine. One published study using ARFI (Tanaka et al., 2011), in attempt to quantify endometrium and myometrium in healthy humans, described difference between the means of the SWV in layers, but the authors reported data as not representative to set a reference range.

In veterinary research, as mentioned previously, studies have described elastography ARFI with significant results, but this is the first study to evaluate the uterine wall parenchyma in animals. The results of the present study suggest that, even though during the postpartum involution the size of the uterus change over the time (Van Wyk et al., 1972a), the elasticity of the uterus undergoes little changes, not being significant. We believe that our study provides important information about the validation of the ARFI technique, and the stiffness of the normal uterus may be useful as a reference parameter for the assessment of uterine integrity during postpartum period.

## Conclusions

Recent studies have reported ultrasonography as a promissory tool to distinguish the pathological from the normal puerperium and thereby avoid unnecessary invasive procedures. In our study, it was possible to describe the physiological changes throughout uterine involution, evaluate the uterine regression development and tissue stiffness during postpartum involution by B mode ultrasonography, color Doppler and ARFI elastography with significant results. We provided valuable information to elucidate the mechanism of physiological uterine involution and uterine characterization during the postpartum period in Santa Ines ewes, to identify and diagnose potential causes of delayed involution. It is also important to mention that the knowledge of the physiological course of uterine regression is a pre-requisite to diagnose pathologies.
